# The Hygiene of the Mouth

**Published:** 1892-10

**Authors:** Charles E. Francis

**Affiliations:** New York


### THE HYGIENE OF THE MOUTH.[1]

BY DR. CHARLES E. FRANCIS, NEW YORK.

It is a fact, apparent to every dentist of much experience, that comparatively few individuals in this country pass the first quarter-century of their existence and possess an unbroken and perfectly healthy denture. Even before their second decade of years has been reached, many individuals have suffered the agonies of odontalgia and lost forever a number of their priceless organs of mastication.

The value of sound teeth to members of the human family can hardly be estimated in way of comparison. Health, comfort, personal appearance, and length of life depend much upon their condition or their integrity. To care for them and to rescue them from the ravages of destructive agents is the mission of our specialty, and to achieve such results we bend our energies and direct our best efforts. It is for this purpose that we come together on occasions like the present. Well-organized societies also exist in every part of the land, where all matters pertaining to our practice can be presented and freely discussed. We listen to carefully-prepared essays on all subjects of interest to our fraternity; we have colleges established in various sections of the country for imparting dental instruction, and text-books teaching principles and methods of practice. We have also numerous literary periodicals teeming

---

[1] Read at a meeting of the American Academy of Dental Science, May 4, 1892.

with rich thoughts and practical suggestions. We furnish our operating-rooms with the most approved appliances and stock our cabinets with the best instruments and choicest materials that have been devised or can be procured, and, indeed, equip ourselves with everything of value that will aid us in our operations and enable us to make such operations successful and satisfactory. It is, in reality, the one great object of our lives to perfect ourselves in the practice of our specialty; yet, with all our prearranged care and painstaking efforts, we find much that is unpleasant to contend against, much to disappoint our hopes and desires, much to undermine and destroy the results of our care and labor,—and it is on this point that I would direct your thoughts on the present occasion.

Patients come to us for attention presenting cases within the oral cavity of every conceivable condition and desire us to restore or repair all defects or deformities that exist therein. Children are brought to us with teeth crowded, irregular, and oftentimes discolored and decayed. We must gain their confidence, relieve them from pain, repair such teeth as are attacked by caries, remove fragments that are a source of irritation, and put their mouths in a condition of cleanliness. We are called upon to expand dwarfed or contracted dental arches, and bring teeth distorted and out of line into a condition of regularity: and all this to insure their preservation and beauty for many years of life in anticipation. And as those of maturer years come to us, other duties are required that likewise tax our time, skill, patience, and energy,—teeth decalcified and broken; crownless roots and jaws almost or quite edentulous; pulps of recent exposure and sickly pulps or pulpless roots. We are confronted with cases of pericementitis and the various stages of alveolar abscess, necrosis of alveolar borders, and sometimes fistulous openings perforating the antrum, recession of gums, absorption of alveolar process, and teeth loosely rocking in diseased sockets. All these and other disordered conditions are presented to the dentist, who is expected to treat them and care for them in an intelligent way and in the best possible manner. Indeed, he is often considered responsible for the preservation and well-being of the teeth of his patients for all future time.

Now, the ideal dentist may have been well trained in the several departments of his calling and proved himself competent to perform each and every operation given to his charge. He may be skilful and painstaking; conscientious in his endeavors to do the best possible thing for his patients, sparing no effort to perfect his operations and to make them a permanent benefit; but can he feel sure that after-results will not disappoint his hopes and that a lapse of time will not exhibit a sad reverse of reasonable expectations? Does the dentist, in reality, do all that is possible to prevent such unfortunate conditions?

Let us suppose that a patient has submitted to the ordeal of having his or her teeth put in what is usually termed "good order." All diseased roots of broken-down teeth have been extracted, every cavity carefully treated and filled, the teeth all around have been well cleansed, and everything so far satisfactory to both dentist and patient. Does this complete the duty of the dentist towards his patient, and is the latter to be dismissed with no word of admonition,—no advice regarding the future care of the teeth, and no instructions for keeping them in a healthy condition and preventing a recurrence of such troubles as have just been corrected? Perhaps many of the gentlemen before me are in the habit of imparting the needed advice to those for whom they operate, but, if statements of the latter can be credited, many dentists are remiss in this duty, and give no word of heed or warning, nor do they picture the serious danger that such neglect incurs.

I believe that hygenic instructions well emphasized, and admonitions equally emphatic, are essentially important not only for the benefit of the patient, but for the good name and professional standing of the dentist as well. And in caring for the teeth, is not the ounce of prevention worth the sixteen of cure?

Every additional year of individual experience and observation impresses me more deeply with the importance of teaching to our clientele lessons of care and cleanliness to their useful organs of mastication. A famous medical writer and lecturer of your city, in one of his lectures on general hygiene, remarked that "if teeth could be kept entirely clean they would never decay." There is certainly a great degree of truth in this statement. We all admit that decalcification of the enamel-structure and destruction of the lime salts of the dentine are due to or hastened by vitiated accumulations lodged about the teeth. But *perfect* cleanliness of the dental organs is a condition which we hardly expect to find in the mouths of our patients; indeed, as a rule, they are very far from showing this pleasing appearance. It is *not* the simple and easy task that many persons suppose to give their teeth the cleansing they need. There are points here and there which escape the searchings of the tooth-brush, and too frequently they look as if a tooth-brush had never come in contact with their surfaces. People generally admire fine-looking and cleanly teeth as they behold them in the mouths of those with whom they meet and converse, and all admit that they add greatly to personal charms, yet few individuals give half the length of time and amount of care actually needed to keep their own teeth in a healthy condition. It is doubtful if, as a rule, when making their own toilet before a mirror, they think of scrutinizing or even looking at their teeth; indeed, it is more than likely that at such times they keep their lips rigidly compressed and have but a faint conception of the condition of the organs hidden beneath. If they give them any thought at all, they content themselves with the belief that they have performed the somewhat irksome duty of brushing them; which duty, however, has been done in so hurried a manner as to have but little beneficial effect.

How painfully discouraging to the dentist, who has taken the utmost pains to nicely prepare cavities and make his fillings compact and durable, to find at a subsequent period his work subjected to the damaging influence of vitiated oral secretions! On the principle that like causes may produce like results, teeth having once been attacked by caries are quite likely to again succumb to the elements of decay where conditions remain unchanged. And where fillings give out or prove failures the dentist is supposed to have done imperfect work, so his reputation suffers to a greater or less extent.

It seems to be one of the most prominent failings of human mortals to saddle the responsibilities of their own sins of omission or carelessness upon the shoulders of others, hence members of our calling get more than their share of blame for the ill results of these neglected duties. We therefore owe it as a duty to ourselves, as well as to our clientele, to urge the latter to take especial pains to keep their teeth in a condition of cleanliness, and instruct them also how they can best accomplish this end.

I am aware of the fact that it is not a particularly pleasant duty to charge our patients with a lack of tidiness, and our criticisms may in some instances be viewed as an ungracious rebuke; nevertheless, as we have chosen the profession that calls upon us to preserve human dentures, why should we not make all reasonable efforts to save them from destruction?

Many individuals with very untidy-looking teeth will assert that they use their tooth-brush two or three times a day. Undoubtedly they *imagine* themselves doing all that is necessary, yet appearances indicate that their efforts are of little account.

Some will excuse neglected duties on the plea of sickness and inability to do themselves justice, but many are proverbially careless or inexcusably negligent, and suffer their teeth to become thickly coated with limy incrustations. As a matter of course, such accumulations are a source of constant irritation to the gums. Wherever they encroach upon the gingival margins congestion follows, and the dental ligament is severed. As recession thus provoked continues, the mischievous deposits increase in quantity and become incrusted beneath the disturbed margins until the alveolar territory is invaded. This induces absorption of the process, the deposits follow, and thus the work of destruction progresses. Any idiopathic tendency to pyorrhetic socket-disease is sure to develop where such conditions are permitted to continue.

When examining the mouths of my patients, I find few, if any, among them with teeth that cannot be improved if cleansed by the dentist. Even though they may appear white and cleanly to the casual beholder, and fairly well at first glance to the dentist, they can be made to look better and feel better after being nicely cleansed. This operation, however, if faithfully done, is much more of an undertaking than is sometimes imagined, requiring greater length of time and far more care than is usually given ; and I feel that we can hardly be too particular in our endeavors to do this work effectually. I have observed that patients appreciate labor thus bestowed upon their teeth more than anything else we can do for them, and are sometimes profuse in expressions of satisfaction with the improved condition of their mouths.

Perhaps my method of cleansing is much the same as adopted by many of the gentlemen before me, yet it may not be out of order to briefly state it. Provided with sickles and scalers of various shapes and sizes, I usually commence by removing the most prominent scales of what is usually denominated, or *misnamed*, "tartar." The patient is supplied with plenty of tepid water for rinsing the mouth at frequent intervals. On the bracket table before me is a glass slab, on one end of which is a small quantity of powdered pumice-stone and borax, of equal parts, mixed. On the other end of the slab are several pellets of cotton about the size of a pea, and compactly rolled. A towel is pinned about the patient's neck to prevent the clothing from getting soiled when rinsing the mouth, or from the powder, should any fall from the brush. With an ordinary pair of tweezers, or gold-carriers, I pick up a pellet of cotton and saturate it with compound tincture of iodine. With this I paint the surfaces of the teeth, three or four at a time, commencing with the incisors, and at the same time, with the fingers of my left hand, keep the lips from coming in direct contact with this application. Then with a small brush fixed in the engine and loaded with the powder, I briskly brush away the stains. Sometimes the engine brushes are rather stiff at first to work well on the broad surfaces of the superior incisors, but they lose much of their rigidity if pressed a few times against the narrower surfaces of the lower incisors. The brush is passed along from tooth to tooth, but frequently removed that the mouth may be rinsed. This process is continued until the stains entirely disappear.

After finishing with the brushes, a moose-head buff is fixed in the engine, with which I go over the teeth to give an extra polish to all surfaces it can be made to reach, and call into service, also, wedge-shaped bits of wood, floss-silk, or wood-fibre, to work between them. Then, with a mouth-syringe, I force tepid water through the dental interstices to clear away the débris. After this washing a careful search is made for such stray atoms of calculus as may hitherto have escaped observation, and when no more can be found, I take a finely-pointed syringe filled with listerine and inject a small quantity between the teeth all around. Where the gums are congested, iodide of zinc may afterwards be applied. It is sometimes advisable for the patient to undergo a second operation; and where socket-disease exists, additional and frequent after-treatment is requisite.

The employment of tincture of iodine greatly facilitates the operation of cleansing, and especially is this the case with children's teeth. It has a wonderful and almost instantaneous effect on the dark stains, combining with the latter, which are rendered so soft as to become easily removed. It also clearly defines the lines of concreted mucus, and enables the operator to readily ascertain if any traces remain undisturbed. It should be applied a number of times during the operation, or until the teeth appear quite cleanly.

Now comes the important question, "How can teeth which we have so carefully treated be kept in the condition we left them?" or, "How can habits of thorough, systematic cleansing be imparted and so impressively taught that instructions may be faithfully carried out or practically demonstrated?" This is a matter that has given me much trouble and anxiety.

I have observed that patients are apt to become interested when we take the trouble to explain to them causes and effects: so during my operations, at convenient intervals, I sometimes give them a brief lecture on dental hygiene. In as concise a manner as possible

I explain the causes of decalcification and of socket-disease, and picture the dire consequences of carelessness and neglect; also tell them how they may lessen the chances of such troubles, and secure to themselves sound teeth and healthy gums. Patients need be taught how to manipulate the tooth-brush, and at what times they can use it to best advantage. I advise them to brush their teeth both at night and in the morning, but to be particularly thorough in doing this just before retiring at night. I remind them of the fact that between the last, or evening meal and the morning repast there intervenes a lapse of about twelve hours, and during most of this time the mouth is comparatively at rest. In the mean time, all particles of food collected about the teeth or filling their interstices are undergoing a process of fermentation.

During sleep the salivary glands are inactive, so no fresh saliva is secreted to dilute the acid formations; hence the acidulated agents work in their slow but sure way night after night for weeks and months, until damaging impressions are made on the tooth-structure. An impression once made is sure to be followed by further invasion, until quite a breach is effected on the calcific territory. We have been told that it is during the hours of sleep that the evil one sows tares, and during the hours of sleep the agents of decalcification do their most effective work. If the teeth are well cleansed at night they require but little brushing next morning. During hours of activity they derive benefit from the friction produced by the mastication of food, while fluids taken into the mouth at various times, and saliva freshly secreted, tend to dilute and check acid fermentation. Whenever I find naturally strong teeth with cavities all along their approximal, buccal, or labial surfaces, I am not surprised when inquiry discloses the fact that remnants of food taken during the day are permitted to remain about them through the night. To be sure, there are individuals with such defective teeth who pretend to use the brush at night, but its use is much like a dash and a promise.

I advise my patients to select brushes small enough to go well into the mouth and reach every possible part of every tooth. Brushes with serrated surfaces will better force the food from between the teeth than will flat-faced ones. The bristles should be stiff enough to be elastic, but not too rigid nor too compactly crowded together, otherwise they will simply glide over the surfaces without entering the interstices. On the other hand, very soft brushes, as of badger- or rabbit-hair, are inefficient and of little value. They possess no elasticity, and come in contact with but little of the surface of each tooth, leaving untouched such places as most need brushing. The same objection will apply to the use of the so-called felt brushes.

In manipulating the brush it is well to adopt some sort of system, and not permit few of the teeth to receive more than their share of attention, while others get little or none. Commence, for instance, with the very back teeth, and work gradually forward towards the centrals; then, with a somewhat quick movement of the wrist, give the brush an occasional semi-rotary motion in a direction *from* the gums. After finishing with one side, treat the other side in the same manner. A suitable dentifrice is essential, which may be improved if the brush is passed across a cake of Castile soap before it receives the powder. On laying aside the brush the mouth should be thoroughly rinsed with water; and finally, some antiseptic agent like listerine, slightly diluted, if allowed to flow around the teeth, will prove beneficial. Such are the instructions given to my patients.

In closing, I must admit that our words of advice are sometimes like seed sown by the wayside, and we get almost discouraged when patients return to us, a few months after their teeth have been well treated, exhibiting sad proofs of negligence. Let us, however, be not weary in well-doing. If we do our part well, we at least have the satisfaction of feeling that we have been faithful to our trust.

